# Cross-sectional study of thyroid associated orbitopathy in a South African thyroid clinic

**DOI:** 10.4102/jcmsa.v3i1.127

**Published:** 2025-02-28

**Authors:** Sandra de Vasconcelos, Hamzah Mustak, Maanda Mabogo, Joel Dave

**Affiliations:** 1 Department of Ophthalmology, Faculty of Surgery, University of Cape Town, Cape Town, South Africa; 2 Department of Ophthalmology, Faculty of Surgery, University of Pretoria, Pretoria, South Africa; 3 Department of Endocrinology, Faculty of Internal Medicine, University of Cape Town, Cape Town, South Africa

**Keywords:** Graves’ disease, ophthalmopathy, prevalence, severity, quality-of-life, EUGOGO, TED-QoL

## Abstract

**Background:**

Thyroid-associated ophthalmopathy (TAO) is a common and debilitating manifestation of Graves’ disease (GD) associated with poor clinical outcomes including impaired quality-of-life (QoL) and socio-economic status. Data on TAO in Africa is scarce and unavailable in the South African population.

**Methods:**

This descriptive cross-sectional study focussed on GD patients attending the thyroid clinic at Groote Schuur Hospital, in Cape Town, South Africa. Participants were evaluated for TAO activity and severity according to the European Group on Graves’ Orbitopathy (EUGOGO) criteria. Quality-of-life was assessed using the TED-QoL questionnaire.

**Results:**

Thyroid-associated ophthalmopathy prevalence was 59.8%. The majority of TAO patients had mild disease (*n* = 73; 59.8%). Based on the severity score alone, moderate to severe disease (*n* = 41; 33.6%) and sight-threatening disease (*n* = 8; 6.6%) were identified and referred for further ophthalmological intervention. There was a statistically significant increase in the QoL score with an increase in the severity score (*p* < 0.05).

**Conclusion:**

The prevalence of orbitopathy was found to be higher than that reported from other continents, but our severity distribution was similar. This study emphasised the importance of evaluating severity as well as the psychosocial aspect of TAO to allow for the identification of and interdisciplinary management of patients to improve patient outcomes.

**Contribution:**

This study was the first to attempt to determine the prevalence and severity of TAO in South Africa. The importance of early TAO detection using both, a clinical and psychosocial tool, is highlighted in this study to reduce the complications associated with TAO.

## Introduction

Thyroid-associated orbitopathy (TAO) is a common autoimmune inflammatory condition of the orbit.^1^ Although its pathophysiology remains unclear, it is known that thyroid stimulating hormone (TSH) receptor antibodies stimulate similar receptors in orbital fibroblasts resulting in inflammatory changes in the extraocular muscles and orbital fat, altering the volume and nature of the orbital soft tissue.^2^

The majority of patients with TAO are hyperthyroid (95.7%) but patients may be euthyroid or hypothyroid (4.3%).^3^ Thyroid-associated ophthalmopathy involves a wide range of ocular changes that usually occur in the setting of Grave’s disease, such as lid retraction, proptosis and motility restriction. Approximately 50% of patients with Graves’ disease (GD) will develop orbitopathy.^4^

Thyroid-associated ophthalmopathy can present with an active phase that subsides to become fibrotic and inactive. The time lapse between the active and inactive phases can range from 6 months to 5 years.^5^

Thyroid-associated ophthalmopathy may be asymmetrical and although most cases do not result in vision loss, exposure keratopathy and optic nerve dysfunction can occur and may be sight-threatening requiring urgent intervention.^6^

The geographically specific pooled prevalence of TAO among patients with Grave’s disease is 40%, with the following geographical breakdown of TAO prevalence – Asia (44%), Europe (38%), North America (27%) and Oceania (58%).^7^ There is a dearth of data from Africa on the prevalence, epidemiology and clinical manifestations of TAO. Specifically, there is no data from South Africa.

Evaluating the activity and severity of orbital changes when diagnosing TAO is essential for management. Treatment decisions are not solely based on the clinical activity; they also consider the severity and duration of TAO, as anti-inflammatory or immunosuppressive treatments are significantly less effective after 18 months of disease duration.^8^

It is important to acknowledge that results from objective disease scoring systems may not correlate with the functional capacity and subjective assessment of disease as experienced by the patient.^9^ A disease-specific questionnaire should be conducted on all patients identified to have TAO. The objective and subjective tools ensure appropriate, timely referral and management.

This study aimed to determine the prevalence, severity and quality-of-life (QoL) in patients attending a tertiary care thyroid clinic with GD complicated with TAO.

## Research methods and design

In this cross-sectional study, patients with GD presenting for their scheduled appointment at the Groote Schuur Hospital thyroid clinic were consecutively sampled. All of the patients attending this specific clinic had been diagnosed by the Endocrinology Department as having GD according to their departmental criteria.

Using a pre-study, estimated prevalence of 50%, with a confidence level of 95% and margin of error of 0.05, the sample size required was 197 patients. Participants were recruited on successive Mondays from April 2021 until the minimum number of 197 participants was reached.

The data collected included demographics (age and sex), clinical activity score (CAS) and severity score as defined by the European Group on GD.^6^

Clinical evaluation of TAO was carried out by assessing for features of active inflammation as well as for the severity of orbital soft tissue changes. The most widely accepted scoring system is the CAS proposed by the European Group on Graves Orbitopathy (EUGOGO). The CAS employs a 7-point scale using classic signs and symptoms of inflammation to detect active TAO. A score of three or greater on the first assessment indicates active disease.

For the classification of TAO severity, the EUGOGO severity score was utilised. Lid retraction was measured using the marginal reflex distance to identify normal, greater or less than 2 mm of retraction. Proptosis was measured using a Hertel’s exophthalmometer and graded as within normal limits, greater or less than 3 mm (normal being less than 20 mm or less than 2 mm difference between eyes).

Visual acuity was measured using a 3 m Snellen chart. Colour vision was assessed using the red saturation test where a red-capped bottle was presented to each eye individually to see if the patient detects a difference in brightness of the red colour between the two eyes.

Screening for a relative afferent pupillary defect (RAPD) was conducted using the swinging flashlight test. Detection of corneal breakdown was conducted using a cobalt blue light after the installation of fluorescein. The severity score was assigned by the researchers according to the results of the above tests, based on the EUGOGO criteria for severity of disease.

The EUGOGO defines mild disease severity as minimal soft tissue changes, lid retraction (< 2 mm) or proptosis (< 3 mm) with little or no extraocular muscle involvement. Moderate to severe TAO consists of some form of active disease with or without ocular motility involvement and inflammatory features interfering with functionality and includes significant proptosis (> 3 mm) and lid retraction (> 2 mm). Serious disease refers to sight-threatening conditions such as corneal breakdown or optic neuropathy.

The TED-QoL questionnaire was used to assess QoL. This three-question questionnaire assessed overall QoL, impact on activities of daily functioning and physical appearance.^10^ The questions required a ranking between 0 (no issues) and 10 (major related issues). Only participants identified with TAO proceeded to complete the QoL survey (TED-QoL). The questionnaire had been formally translated and was completed by participants in their language of preference (English, Afrikaans or isiXhosa). The medians per question were compared between the three severity groups using non-parametric Kruskal–Wallis tests.

Non-parametric Kruskal–Wallis tests were used to compare scores between groups, and Kendal’s tau and Spearman’s rank correlations were used to assess ordinal by ordinal associations.

### Ethical considerations

This study was undertaken in accordance with the guidelines of the Declaration of Helsinki and was approved by the Human Research Ethics Committee of the Faculty of Health Sciences of the University of Cape Town (HREC 095/2021). Prior to participating in the study, procedures and risks were explained to the participants, all of whom gave written informed consent.

## Results

A total of 204 participants gave written informed consent and completed the data collection; however, four were excluded because of having other ophthalmological conditions which would influence the severity scores.

### Demographics

The majority of the participants in the study were female (*n* = 174; 85.3%) and of the patients with TAO, 87% were female with a female : male ratio (6.6:1).

A total of 66.3% of the participants with TAO were in the 31–50 year age group. This is in keeping with the EUGOGO studies showing an age range of 34–62 years (mean of 48).

### Prevalence, activity and severity

Of the 200 included participants, 122 had TAO, resulting in a prevalence of 59.8% (95% confidence interval [CI]: 52.1–66.5).

The prevalence of CAS signs is shown in Table 1. The median CAS score was 0 with a range of 0 to 4 and an interquartile range from 0 to 2. Within the participant sample, 71 (58%) patients had a score of 0.

**TABLE 1:** Clinical activity score breakdown in the patient cohort (*N* = 200).

CAS criteria	*n*	%
Retrobulbar pain	29	23.8
Pain on extraocular movement	6	4.9
Eyelid redness	3	2.5
Conjunctival redness	16	13.1
Eyelid swelling	24	19.7
Caruncle swelling	12	9.8
Chemosis	4	3.3

CAS, clinical activity score.

The prevalence of the severity signs in the 122 participants with TAO is shown in Table 2.

**TABLE 2:** Prevalence of severity signs (*n* = 122).

Severity score signs	*n*	%
**Eyelid retract**		
< 2 mm	40	32.8
> 2 mm	40	32.8
None	42	34.4
**Exophthalmos**		
< 3 mm	51	41.8
> 3 mm	32	26.2
None	39	32.0
**Soft tissue**		
No	42	34.4
Yes – mild	68	55.7
Yes – moderate	12	9.8
**Diplopia**		
No	116	95.1
Yes	6	4.9
**Corneal signs**		
No	116	95.1
Yes	6	4.9
**Optic nerve dysfunction**		
No	119	97.5
Yes	3	2.5

The median severity score was 1 with an interquartile range of 1–2. The majority of participants with TAO were graded as having mild disease. Notably, 33.6% of participants with TAO had moderate to severe disease with 6.6% having sight-threatening disease (Table 3).

**TABLE 3:** Frequency of thyroid-associated ophthalmopathy severity.

Severity score	Frequency (*n*)	%
Mild	73	59.8
Moderate to severe	41	33.6
Sight threatening	8	6.6

**Total**	**122**	**100.0**

### Quality-of-life assessment

The median score for ‘overall quality of life’ was 4 out of 10, for ‘interference with activities of daily living’ was 2 out of 10 and for ‘overall satisfaction with appearance’ was 6 out of 10.

**FIGURE 1:**
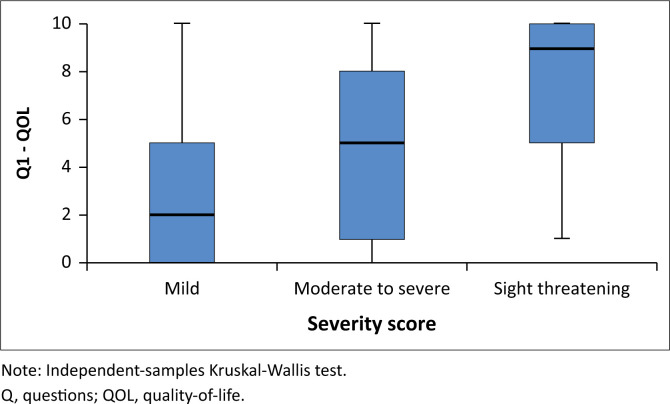
Quality-of-life question one – Impact of thyroid eye disease on overall quality-of-life according to thyroid-associated ophthalmopathy severity.

**FIGURE 2:**
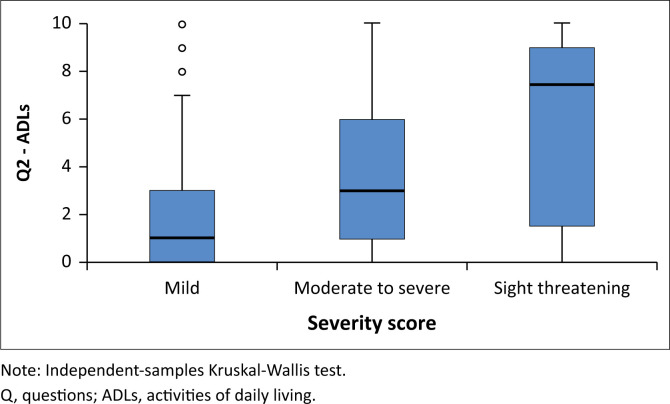
Quality-of-life question two – Impact of thyroid eye disease on the ability to carry out activities of daily living according to thyroid-associated ophthalmopathy severity.

**FIGURE 3:**
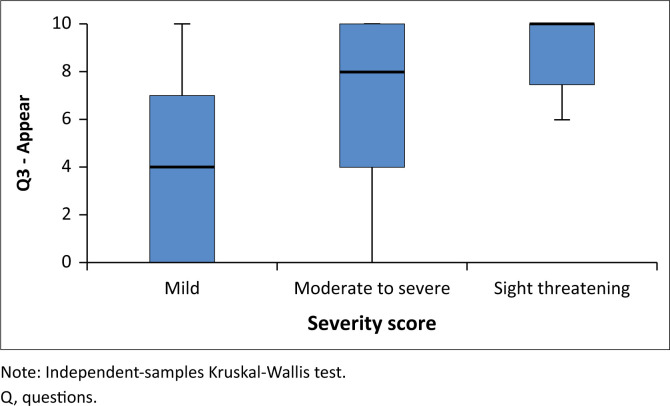
Quality-of-life question three – Impact of thyroid eye disease on patient satisfaction with appearance according to thyroid-associated ophthalmopathy severity.

When stratified by severity score, there was a clear trend of increasing QoL scores with increasing severity scores. Higher QoL scores were associated with greater dissatisfaction by participants. For all three questions, there was a statistically significant increase in QoL score with an increase in severity score (see Figure 1, Figure 2 and Figure 3).

## Discussion

The majority of TAO patients in this study were female in the age range of 31–50 years, mirroring the trends described in other epidemiological studies.^6,7,11,12^ Although this study did not include ethnicity, multiple studies have shown that ethnicity is not associated with the development of TAO. Stein et al. found no difference among white, black, Latino or Asian American patients.^13^ Similarly, Lim et al. demonstrated that ethnicity did not play a role in the development of TAO.^14^ Cape Town is known to have a rich genetic ancestry with a large mixed-race population making it difficult to categorise participants into traditional ethnic categories.

Our study found a TAO prevalence of 59.8% (95% CI: 52.1–66.5). When compared to the meta-analysis conducted by Chin et al. which pooled 33 articles and found that European prevalence was 38%, Asian prevalence was 44% and North American prevalence was only 27%.^7^ With no African studies included in this meta-analysis our prevalence is higher.

When comparing to African studies, Ogun and Adeleye’s study to determine the prevalence of TAO in Nigerians attending an endocrine clinic found that ≥ 50% of patients with GD had orbital complications, with the majority found to have mild disease.^15^

Ackuaku-Dogbe et al. conducted a study in a tertiary facility in Accra, Ghana which found the prevalence of TAO to be 60.3%. There was a female predominance, and the majority had mild disease (64.96%).^12^

The prevalence from Ackuaku-Dogbe et al. in Ghana and our South African study was higher than that described by Okafor et al. in Nigeria who found the prevalence of TAO in their setting to be 32%.^16^ The average prevalence calculated from these three African studies would be 50.5% which is still higher than the prevalence in other continents.

With a higher African prevalence, this could suggest that there is a geographical difference in the manifestation of thyroid eye disease when compared to other areas like Europe or North America.

The majority (95.1%) of patients in our cohort had clinically inactive TAO, with 58% (*n* = 71) scoring 0 out of 7 on the CAS. An initial first assessment score of three or greater was only found in 4.9% of patients.

Participants’ TAO was graded using the EUGOGO severity criteria as being mild (59.8%) moderate-to-severe (33.3%) and sight-threatening (6.6%).

There is always a concern when comparing developed countries with developing countries as the profile of disease presentation may be different. Results from Perros et al.’s EUGOGO position statement stated that 65% of all cases of TAO were found to have mild TAO, 33% moderate to severe and about 2% sight-threatening disease.^11^ Although our TAO prevalence is higher than those found on other continents our severity distribution of mild, moderate-to-severe and sight threatening is similar.

Having identified the activity and severity of the TAO, an assessment of the impact of this disease on the patient’s QoL should be obtained through a disease-specific questionnaire.

To evaluate the patients’ subjective view of the disease, various questionnaires have been developed and validated. Terwee et al.^17^ developed a graves orbitopathy-specific quality-of-life questionnaire (GO–QOL). The 16-question disease-specific questionnaire required patients to evaluate the disease’s effect on visual functioning and the psychosocial consequences of their change in appearance as a result of the disease.^17^ Although the GO-QOL has been successfully translated into multiple languages, there is concern about translating phrases and the cross-cultural equivalence of this translation. Cultural diversity and idiosyncrasies result in the importance and meaning of certain questions being lost. Patients cannot complete the questionnaire as certain questions are not culturally important (i.e. riding a bicycle) which might yield an incomplete or false result.^18^ The GO-QOL with a total of 16 questions may be quite cumbersome for routine assessment in a busy clinic setting.

This led to Fayers et al. developing a questionnaire in which QoL was assessed using only three questions. The simplified three-question survey (TED-QoL) dealt with three psychosocial aspects of TAO – the patient’s QoL; activities of daily living and satisfaction with their appearance. The study showed the TED-QOL tool was significantly faster to complete, and had higher rates of completion with a similar validity and reliability to the longer questionnaire (GO-QoL) available from Terwee et al.^10^

Son et al. translated and applied the TED-QOL questionnaire to a Korean population and found it had a good correlation with clinical severity and was comparable with GO-QOL. It was also concluded that because of the simplicity of the questions, this tool could be used in different populations as cross-cultural translation was possible. The ease of scoring results leads to the rapid identification of patients needing psychosocial support and referral.^18^

A statistically significant increase in the QoL score was noted with an increasing severity score. This validates the questionnaire and speaks to the patient’s need for intervention and referral for psychosocial and surgical intervention. Although this three-question questionnaire is much simpler than the traditional GO-QOL questionnaire, it serves as a good screening tool to identify patients who require a more multidisciplinary approach from the endocrinology clinic and should weigh in on further management and referral.

Most patients with mild TAO experience spontaneous resolution of eye manifestations so a ‘watch and wait’ strategy with local treatments may be sufficient, but those mild TAO patients with markedly impaired QoL can be considered for low-dose immune suppression or surgery as per the latest EUGOGO guidelines.^6^ The QoL questionnaire is thus fundamental in deciding whether treatments employed for moderate-to-severe TAO can be justified in patients with mild TAO if their QoL is affected significantly.

Moderate to severe TAO requires active management to shorten the active phase of the disease. Immunosuppressive agents may improve both subjective and objective eye manifestations especially if initiated within 1 year of its onset.^6^ It is essential to start treating the thyroid dysfunction and the TAO concurrently with immunosuppression and anti-thyroid drugs so early referral of these patients is important. Sight-threatening diseases require immediate, same-day referral for intervention.

In our study based on severity score alone, 40.2% of the patients with TAO would require referral for further ophthalmological assessment and intervention as they were graded as having moderate-to-severe or sight-threatening TAO. The ophthalmological assessment will ensure that patients get immune suppression early to prevent progression or if in the fibrotic phase can start to plan for the staged surgeries needed to restore the eye anatomy and improve QoL.

If only CAS is carried out without a formal severity and QoL score, patients requiring referral and intervention will be missed. This study would advocate for GD patients to have a CAS, severity score and QoL questionnaire conducted by the primary treating physician, in the first consultation, to identify patients for referral early. The scoring system provides an objective baseline for repeated severity scoring at follow-up visits.

### Limitations

This study was the first to attempt to determine the prevalence and severity of TAO in a South African population. Our study may however not be a true reflection of the rest of the country because of selection bias as this study was conducted out of a tertiary facility.

A confounder could be that the screening was conducted by two researchers who have an established background in Ophthalmology and as such the results of the CAS and Severity Score may have differed if carried out by a non-ophthalmologically trained individual.

The low CAS score and severity grading might have been impacted by the fact that 84% of the patients enrolled in the study were attending follow-up appointments with prior initiation medication or surgical intervention. It was not documented if the patient was euthyroid or dysthyroid when conducting the activity and severity score. When comparing our severity distribution of mild, moderate-to-severe and sight-threatening disease to international studies, our result were comparable.^11^

Other limitations include the cross-sectional nature of this study with a lack of follow-up – a prospective study would have given more insight into severity, progression and regression. A multi-centre prospective study with a larger sample size with follow-up severity and QoL scoring would better clarify the natural history of TAO in South Africa.

## Conclusion

This study was to the best of our knowledge the first of its kind to be undertaken in a South African population. Thyroid-associated ophthalmopathy prevalence is higher than those found on other continents, but the severity distribution is similar, with few clinically active and sight threatening cases. There is a role for the use of the EUGOGO CAS and severity scale to be used in conjunction with a QoL questionnaire to identify patients in need of earlier ophthalmological intervention. Early TAO detection through active screening using a clinical and psychosocial tool would help to reduce the progression of TAO and its associated complications. A prospective study would help to better define the natural history of TAO in an African population.
